# Hybrid Molecules as Potential Drugs for the Treatment of HIV: Design and Applications

**DOI:** 10.3390/ph15091092

**Published:** 2022-08-31

**Authors:** Wissal Liman, Nouhaila Ait Lahcen, Mehdi Oubahmane, Ismail Hdoufane, Driss Cherqaoui, Rachid Daoud, Achraf El Allali

**Affiliations:** 1 African Genome Center, Mohammed VI Polytechnic University, Ben Guerir 43150, Morocco; 2 Department of Chemistry, Faculty of Sciences Semlalia, BP 2390, Marrakech 40000, Morocco

**Keywords:** anti-HIV, hybrid compounds, multi-targeting, reverse transcriptase, integrase, protease, molecular docking, MD simulations, pharmacophore, structure activity relationship

## Abstract

Human immunodeficiency virus (HIV) infection is a major problem for humanity because HIV is constantly changing and developing resistance to current drugs. This necessitates the development of new anti-HIV drugs that take new approaches to combat an ever-evolving virus. One of the promising alternatives to combination antiretroviral therapy (cART) is the molecular hybrid strategy, in which two or more pharmacophore units of bioactive scaffolds are combined into a single molecular structure. These hybrid structures have the potential to have higher efficacy and lower toxicity than their parent molecules. Given the potential advantages of the hybrid molecular approach, the development and synthesis of these compounds are of great importance in anti-HIV drug discovery. This review focuses on the recent development of hybrid compounds targeting integrase (IN), reverse transcriptase (RT), and protease (PR) proteins and provides a brief description of their chemical structures, structure–activity relationship, and binding mode.

## 1. Introduction

Human immunodeficiency virus (HIV) infection poses a serious global public health threat [1]. It is a lentivirus that spreads through vaginal fluids and blood and infects the immune system, especially CD4 T cells, reducing the ability of the immune system to fight infection. The chronic decline in CD4 T cells caused by HIV infection leads to acquired immunodeficiency syndrome (AIDS, also known as stage 3 HIV) [2,3]. According to the Global HIV & AIDS statistics report of 2020, 36.3 million people have died since the beginning of the pandemic. Currently, 37.7 million people are living with HIV worldwide, only 75% of whom have access to antiretroviral therapy (ART) [4]. This means that the remaining 25% (~9.5 million) are at risk of progressive immune system deterioration, making them vulnerable to life-threatening infections and cancer if not treated early. Despite advances in treatment and prevention strategies that have led to an increase in the average age of death of HIV-positive people and a decrease in the transmission rate of the virus compared with recent years, the virus continues to claim lives: 680,000 people have died as a result of AIDS-related diseases and more than 1.5 million people became newly infected in 2020 [4]. To date, combination antiretroviral therapy (cART), also called highly active antiretroviral therapy (HAART), is considered the most potent treatment for HIV-positive patients [5,6]. This therapy combines two or more drugs to target one or more viral proteins [7]. Despite its effectiveness in controlling disease progression and survival of HIV-infected patients, cART suffers from at least one important problem, such as incomplete efficacy, drug toxicity, high cost, and the emergence of viral drug resistance [8]. Therefore, it is crucial to design and synthesize new drugs with unique therapeutic strategies. The molecular hybrid approach is a promising innovative approach used to combat infectious diseases and circumvent existing obstacles. The molecular hybrid approach is a strategy in which two or more pharmacophores of bioactive scaffolds are combined to form a single molecular structure with higher affinity and activity compared to their parent molecules [9]. This approach has enabled the development of new drugs with improved biological potential and reduced side effects, especially in cancer treatment. In addition, interesting hybrid anti-HIV agents targeting proteins integrase (IN), protease (PR), and reverse transcriptase (RT) are currently being developed. In this review, recent remarkable efforts in the discovery of hybrid molecules with promising anti-HIV activity are highlighted and their structure–activity relationship is briefly explained.

## 2. Hybrid Drugs: Concept and Approaches

**Figure 1:**
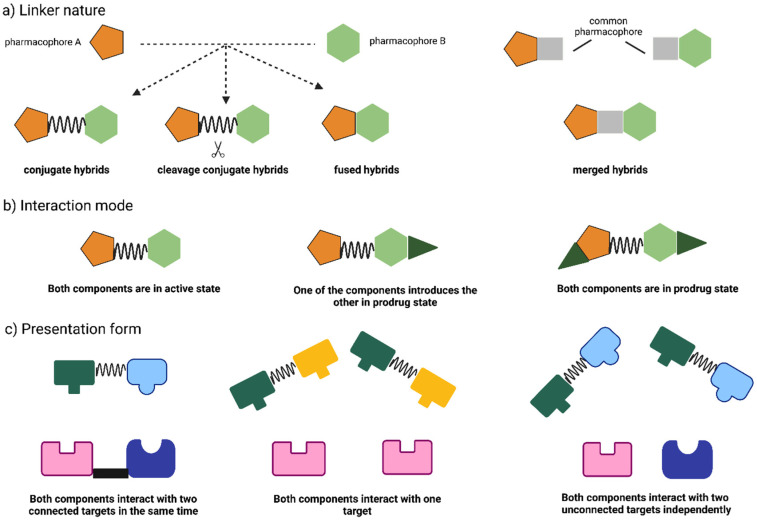
The classification of hybrid compounds is based on: (**a**) The nature of the linker used; (**b**) The mode of the interaction of each pharmacophore with targets; (**c**) The form of presentation.

The concept of hybrid drugs usually involves the combination of two or more pharmacophores of bioactive scaffolds in a single molecular structure [10,11]. Hybrid molecules are categorized according to the type of linker used to combine the two pharmacophores, the mode of the interaction of each pharmacophore with the targets, and the form of their presentation [12,13,14], as shown in Figure 1. The characteristics of the linker used to bind both pharmacophores could be a metabolically stable linker that is not a component of either pharmacophoric units (conjugate hybrids), or a metabolically cleavable linker that is metabolized in the biological system to release the two pharmacophoric units that interact independently with different targets (cleavage conjugate hybrids). The merged hybrids are molecules that have their frameworks integrated by utilizing the shared pharmacophore in the initial structures. The fused hybrids are molecules in which the linker has been reduced in size so that the pharmacophore units are in contact. There are many modes of interaction with the target for hybrid drugs. In the first scenario, both pharmacophore units interact with the same target. A second situation is when the two pharmacophore units act independently on two unrelated targets, while the third situation is when both pharmacophore units act simultaneously on two related targets. The hybrid molecule can be presented in a variety of forms. In the first case, both components can be delivered in the active form. In the second case, one of the components is used to provide the other component in prodrug form, and in the third case, both components are provided in prodrug form.

## 3. HIV Replication Cycle and Inhibition Sites

**Figure 2:**
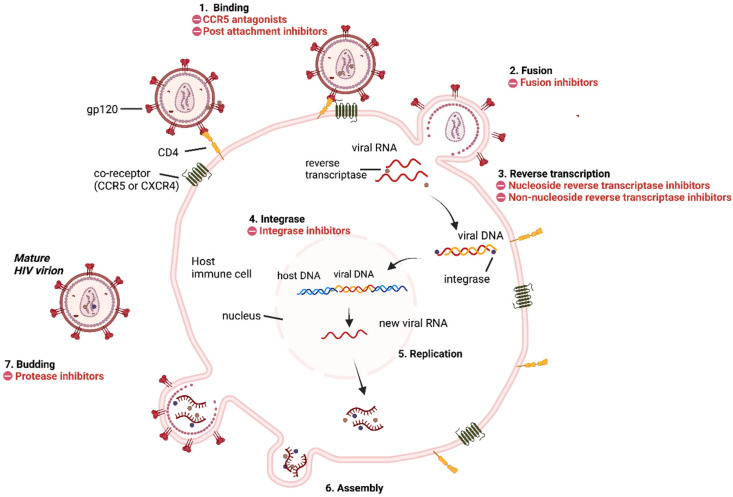
Simplified representation of the HIV life cycle mapping the types of inhibitors that can interrupt viral entry and replication.

HIV, like other retroviruses, has a spherical shape and a diameter of approximately 120 nm. It is composed of two copies of single-stranded RNA in the positive sense, which are encased in a nucleocapsid protein. When a newly produced virus particle buds from the cell, it is wrapped by the viral envelope, which consists of phospholipids derived from the membrane of a human cell. The envelope is a trimeric protein with three cap-like glycoprotein 120 (gp120) subunits and three stem-like glycoprotein 41 (gp41) subunits that secure the envelope in the viral membrane [15,16]. This glycoprotein complex enables the virus to bind and fuse with target cells, allowing the infection cycle to begin. HIV infection is often divided into seven distinct phases that begin with the binding of the virus to the host cell and end by forming new infectious HIV virions [17] (Figure 2). HIV penetrates cells via binding of the viral surface glycoproteins gp120 to the primary host cell receptor CD4 and the cellular co-receptors C-X-C motif chemokine receptor type 4 (CXCR4) or chemokine receptor type 5 (CCR5) [18,19]. This binding causes a conformational shift that allows gp41 to breach the cell membrane. The viral and cellular membranes can then fuse together and the viral core is uncoated, releasing the viral genome and enzyme (Ribonuclease H, RNas H) essential for the reverse transcription of RNA into double-stranded DNA, allowing complementary DNA (cDNA) to form [20,21]. Subsequently, the cDNA is transcribed again into double-stranded DNA (dsDNA), which, together with integrase and auxiliary proteins, forms the pre-integration complex (PIC). An active mechanism transports this PIC from the cytoplasm via the nuclear pore complex into the nucleus [22]. Subsequently, the integrase enzyme integrates the DNA of the virus into the genome of the host cell once it enters the nucleus [23]. The integrated viral genome then uses the machinery of the infected host cell to produce long chains of viral proteins. After viral protein synthesis, the viral genomic RNA and proteins are brought to the plasma membrane and assembled into new immature viral particles, resulting in the budding and release of immature virions [24]. The newly released viral particles push out of the host cell and then the enzyme protease of the virus cleaves the gag-pol polyprotein, producing a mature and infectious viral particle [25].

**Figure 3:**
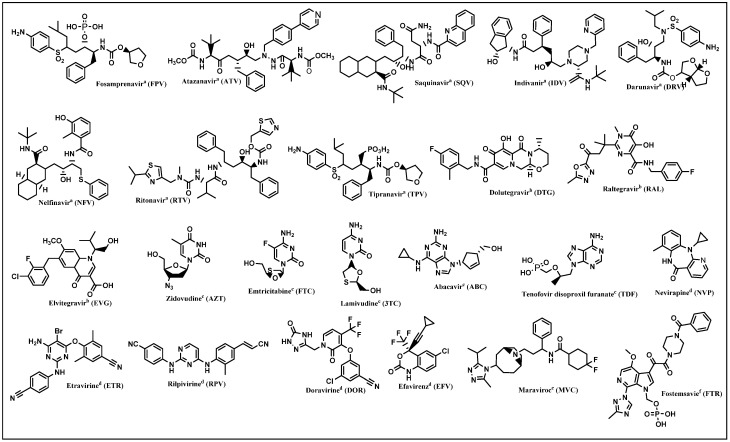
ART drugs approved by the FDA (a = PIs, b = INSTIs, c = NRTIs, d = NNRTIs, e = CCR5 receptor antagonist, f = post-attachment inhibitors).

The life cycle of HIV provides information about how the virus attacks human cells and how it replicates itself. This helps scientists develop an effective treatment to suppress HIV by reducing the viral load. To date, the FDA has approved over 40 ART and cART drugs [26]. The drugs from ART are classified into seven classes according to their mode of action in the HIV life cycle: CCR5 receptor antagonists, post-attachment inhibitors, protease inhibitors (PIs), integrase strand transfer inhibitors (INSTIs), nucleoside reverse transcription inhibitors (NRTIs), non-nucleoside reverse transcription inhibitors (NNRTIs), and fusion inhibitors (Figure 3). The cART drugs are combination treatments of two to five ARTs targeting one or more viral proteins (Table 1). Despite the great strides that current treatments have made in terms of survival and quality of life for HIV-positive persons, resistance to these drugs limits their efficacy in a growing number of HIV-infected individuals. It is therefore critical to develop new anti-HIV drugs with a novel therapeutic mechanism. Hybrid molecules can solve the problem of drug resistance by inhibiting multiple targets simultaneously and by protecting each other’s pharmacophore components. Thus, these hybrids might target the virus through two different pathways, which would prevent or delay the emergence of resistance [9,27]. In addition, hybrid molecules improve drug efficacy and reduce side effects and manufacturing costs. Most hybrid molecules developed target the proteins RT, IN, and PR, as they play promising key roles in HIV replication. 

## 4. Hybrids Acting on Selected Targets

### 4.1. RT/PR Inhibition

The combination of RT inhibitors with PR inhibitors has recently become standard clinical practice to obtain sustained benefits from antiviral therapy, which helps prevent the emergence of viral resistance and reduces the side effects of chemotherapy. 

#### 4.1.1. Cinnamic and Phenylpropionic Amide Derivatives

**Figure 4:**
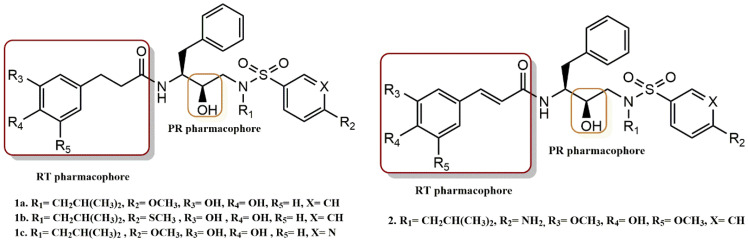
RT and PR pharmacophores of new active synthesized compounds **1a-c** and **2**.

Zhu et al. developed and evaluated a new class of HIV-1 PR/RT dual inhibitors based on possible ligand-binding site interactions and uniformity of key residues in the active sites [28]. Among these inhibitors, compounds **1a**, **1b**, **1c** and **2** showed the highest activity (Figure 4). The molecular modeling study for inhibitors **1a**, **1b**, and **1c** was performed to investigate potential hydrogen bonding interactions and hydrophobic contacts. With a binding value of −13.54 kcal/mol, inhibitor **1a** fit quite well into the HIV-1 PR binding site. Inhibitor **1b** also fit well into the protease cavity but had a weaker negative binding value (−12.18 kcal/mol) than inhibitor **1a**, indicating poorer binding with the enzyme, consistent with its PR inhibitory activity. When inhibitor **1c** was introduced into the active cavity of the protease, it formed several strong hydrogen bonds with PR. To verify the effectiveness of the ligands’ druggability and their binding mechanism, molecular dynamics (MD) simulations were performed for complexes **1b**/PR, **1b**/RT, **1c**/PR, and **1c**/RT. The RMSD analysis revealed that during the simulation, the binding mode for all four complexes barely altered. However, it supported the fundamental tenet of dual inhibitor design by pointing out that the initial docked binding modes may represent interactions between the inhibitors and the active sites of HIV-1 PR or RT. Similarly, the MMPBSA method was used to assess the binding energies for the complexes **1b**/PR, **1b**/RT, **1c**/PR, and **1c**/RT based on the MD simulation trajectory. Based on this, both **1b** and **1c** (which had computed DGs of −11.59 and −13.88 kcal/mol, respectively) demonstrated high binding affinity with protease. While **1b**, **1c**, and RT were anticipated to have binding free energies of −8.37 and −8.75 kcal/mol, respectively, the other three did not. As a result, the MD findings suggest that **1b** and **1c** would bind to the dual targets (PR and RT).

In vitro assays were performed for all inhibitors using the FRET-based protease activity assay and HIV-1 RT activity. In addition, compounds **2** and **1b** were tested in late and early-stage HIV-1 life cycle assays, with DRV and EFV serving as controls to further confirm the dual inhibitor targets. Inhibitor **1b** showed an antiviral EC_50_ of 2.31 µM and 97% inhibition in late-stage HIV-1 at a concentration of 10 µM and an EC_50_ of 8.32 µM and 68% inhibition in early-stage HIV-1. Inhibitor **2** showed an antiviral EC_50_ value of 37.35 µM and 30% inhibition in late-stage HIV-1 and an EC_50_ of 68.02 µM and 14% inhibition in early-stage HIV-1.

#### 4.1.2. Coumarin Derivatives

**Figure 5:**
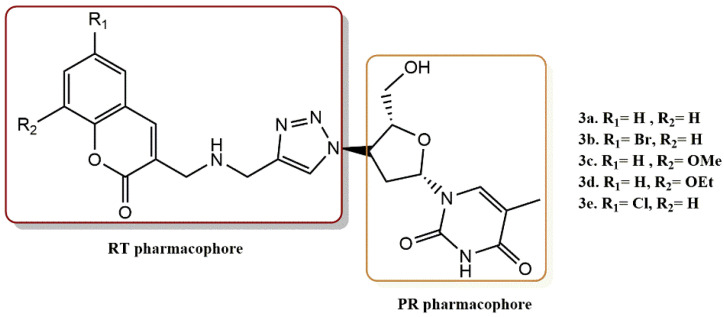
RT and PR pharmacophores of new active synthesized compounds **3a-e**.

Olomola et al. reported the employment of 3-(chloromethyl)-coumarins from the Baylis–Hillman reaction to create a series of compounds (**3a-e**) that include triazolothymidine and coumarin moieties as possible PR and RT dual-action HIV-1 inhibitors as well as scaffolds for further structural development (Figure 5) [29]. To study the interactions between these substances and the receptor sites of the HIV-1 PR and RT enzymes, docking experiments were carried out. The docking of compound **4a** to the HIV-1 PR receptor site indicated putative hydrogen bonding interactions between the coumarin and AZT moieties and the close-by protein residues. Regarding the range of accepted structural motifs, the non-nucleoside binding pocket of HIV-1 RT seemed to be rather flexible. When the ligands **3a-e** were docked to this pocket, it was evident that they might form hydrogen bonds with the amino acid residues Lys101, Lys172, Ile178, Ile180, Ile1135, Glu1138, and Thr1139 that border the pocket. 

**Figure 6:**
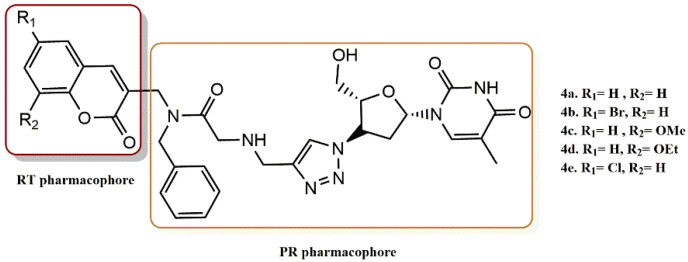
RT and PR pharmacophores of new active synthesized compounds **4a-e**.

Olomola et al. successfully reported the efficient synthesis of a series of N-benzylated coumarin-AZT conjugates [30]. The ability of the ligands (**4a-e**) to decrease the activity of both enzymes was tested in order to identify potential dual-acting inhibitors of HIV-1 PR and HIV-1 RT (Figure 6). The inhibitory activity of the ligands as HIV-1 PR and RT inhibitors was evaluated in contrast to ritonavir and AZT using commercial HIV-1 PR28 and HIV-1 RT29 assays. Since all five ligands (**4a-e**) display considerably greater IC_50_ values than AZT, it appears that the N-benzyl group added to improve the inhibition of HIV-1 PR has substantially increased the inhibitory activity of HIV-1 RT instead. Using docking studies, the interactions between the unsubstituted bifunctional ligand **4a** and the HIV-1 PR and NNRT enzyme receptor sites were investigated. The N-benzylated coumarin-AZT compound **4a** was docked to the active sites of HIV-1 PR and HIV-1 NNRT. According to the findings, it has good affinity for the HIV-1 PR active site and is capable of a variety of hydrogen interactions with the residues Arg8, Gly27, Asp29, Asp30, Gly48, Gly49, and Ile50 via its coumarin and AZT moieties. The intriguing enzyme inhibitory activity of N-benzylated coumarin-AZT conjugates **4a-e** on HIV-1 RT and the potential hydrogen bonding interactions with amino acid residues at the receptor site may be explained by this arrangement.

**Figure 7:**
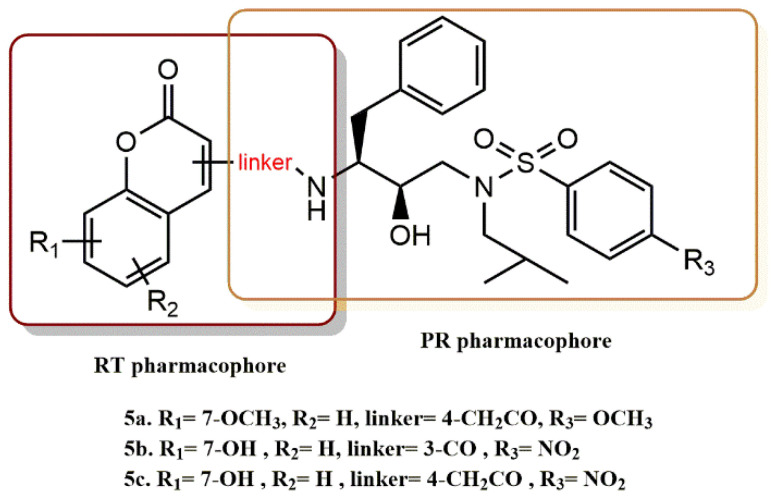
RT and PR pharmacophores of new active synthesized compounds **5a-c**.

Zhu et al. designed and synthesized new coumarin derivatives characterized by different linkers showing excellent activity against PR and weak inhibition of RT [31]. Coumarin moieties were introduced into HIV-1 protease inhibitors, leading to the development of new fusion-type pharmacophore inhibitors containing a coumarin moiety. Compounds **5a** and **5b** (Figure 7) from this group of inhibitors inhibited PR with IC_50_ values of 15.5 and 62.1 nM, respectively, and also had a negligible effect on RT with IC_50_ values of 241.8 and 188.7 µM, respectively, suggesting the potential for future development of dual HIV-1 PR/RT inhibitors. Using a FRET, all recently synthesized substances were evaluated for their in vitro PR activity against the HIV-1 wild-type protease. Cell-based experiments were used to further evaluate the chosen inhibitors. Molecular docking experiments on inhibitor **6c**, which displays both very powerful action against PR and less active RT inhibition, were carried out to investigate the antiviral activities of the inhibitors. Using the crystal structures of the HIV-1 PR and RT, molecular docking was used to examine the common mechanism of inhibitory binding. Significantly, hydrogen bonds and van der Waals interactions allowed inhibitor **5c** to fit neatly into the PR binding site. Thus, multiple hydrogen bonds between residues Arg8 and Gly48 and van der Waals interactions with the outer enzyme atoms are possible, both of which could be responsible for the promising HIV-1 PR inhibitory activity. In contrast, compound **5c** with a 2-oxo-2H-chromium moiety could not be placed directly in the tight pocket of RT by about thirty thousand-fold compared to the potency of PR. Although unsatisfactory, the poor activity against RT might be attributed to the hydrogen bonding interaction between Lys223 and other residues. To better explain the activity of this type of dual inhibitors, the coumarin moieties that sterically prevent adaptation to the RT active site could exert their inhibitory effect on RT by removing the bulkier furanone ring and replacing it with a smaller, flexible chain, which would not affect the activity against PR according to the molecular docking studies. Molecular docking studies suggest that the 2-oxo-2H-chromen of coumarin units may present a steric hindrance to their incorporation into the tight active site pocket of RT. Removal of the bulky furanone ring and its replacement with a flexible miniature linker could exert the inhibitory effect of RT and enhance its activity against PR.

#### 4.1.3. Fullerene Derivatives

There are limited examples of fullerene derivatives’ synthetic history; however, several groups have reported on 3D QSAR, molecular docking, and MD modeling of fullerene-based HIV-1 PR inhibitors [32,33]. 

**Figure 8:**
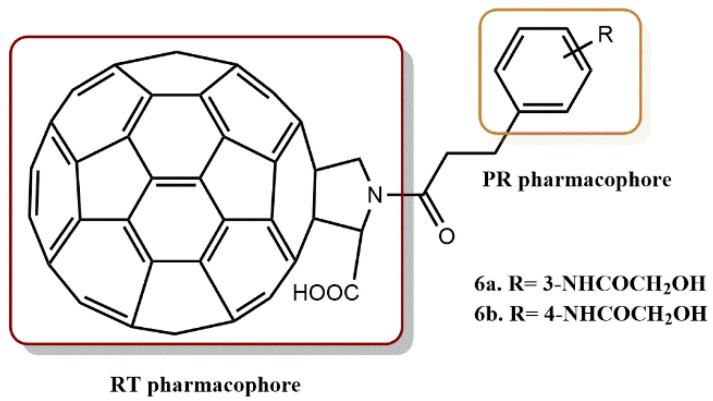
RT and PR pharmacophores of new active synthesized compounds **6a** and **6b**.

In a docking simulation, Ibrahim et al. and Saleh et al. found that fullerene derivatives with a hydroxymethylcarbonyl (HMC) moiety, the transition state of substrate processing, could exhibit high HIV-1 PR inhibitory activity as a result of the HMC moiety’s efficient interaction with the HIV-1 PR aspartic acid residues [34,35]. In this context, Takumi et al. developed three distinct types of novel fullerene derivatives: those of the piperidinium, pyridinium, and proline types [36]. Nine compounds were shown to inhibit HIV-1 RT and PR among the compounds investigated, with IC_50_ values in the low micromolar range. Using HIV-1 recombinant RT, the fullerene derivatives’ HIV-1 RT inhibitory effects were initially investigated. All of the new fullerene compounds created in this work have HIV-1 RT inhibitory properties that were more potent than nevirapine. Recombinant HIV-1 PR was used to evaluate the HIV-1 PR inhibitory action. Proline derivatives **6a** and **6b** (Figure 8) displayed the most balanced activity out of all these derivatives, exhibiting comparable levels of inhibitory activity against HIV-1 RT and HIV-1 PR with submicromolar IC_50_ values, indicating that they may effectively inhibit HIV replication through a synergistic effect as a dual inhibitor for HIV-1 RT and HIV-1 PR. 

**Figure 9:**
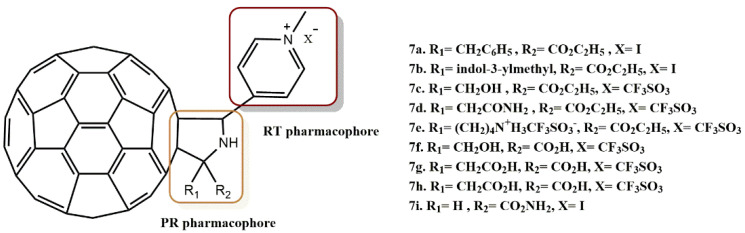
RT and PR pharmacophores of new active synthesized compounds **7a-i**.

Kobayashi et al. reported the synthesis of four types of novel fullerene derivatives: Pyridinium/ethyl ester-type derivatives, Pyridinium/carboxylic acid-type derivatives, Pyridinium/amide-type derivatives, and Pyridinium/2-morpholinone-type derivatives [37]. Of the compounds studied, **7a-i** (Figure 9) inhibited HIV-1 RT and PR, having an IC_50_ values in the micromolar range. 

#### 4.1.4. AZT Derivatives

**Figure 10:**
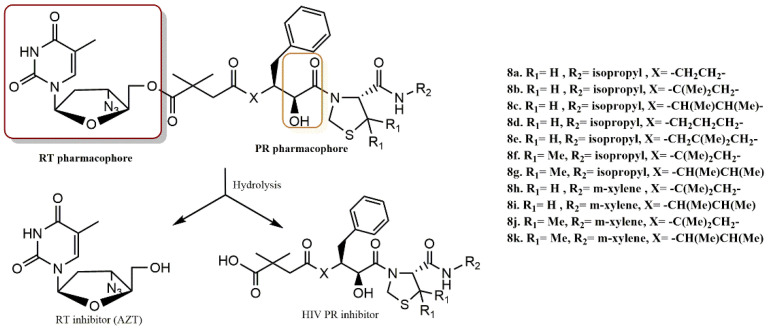
RT and PR pharmacophores of new active synthesized compounds **8a-k**.

Matsumoto et al. developed and synthesized a series of modest inhibitors (**8a-k**) with the hydrophilic carboxyl group based on the idea of emulating the substrate transition states of HIV protease (Figure 10) [38]. Evaluation of the augmentation of anti-HIV activity was done using the relative potency based on the EC_50_ of AZT. All conjugates with the exception of substances **8d** and **8e** had strong anti-HIV activity and minimal cytotoxicity. Compounds **8g**, **8i**, and **k** displayed antiviral activity that was comparable to AZT. Compound **8f** (EC_50_ = 10 nM), which was 6.6 times more effective than AZT, showed excellent anti-HIV efficacy. Among these conjugates, Compound **8j** has shown extraordinarily powerful antiviral activity that was 46-fold greater than that of AZT. Investigations were also conducted into the pharmacodynamic characteristics, including the stability of the conjugates in aqueous medium, rat plasma, and human serum. These conjugates’ synergistic improvement of anti-HIV activity may be due to their capacity to penetrate the target cell and then renew two separate classes of anti-HIV agents in the cytoplasm.

**Figure 11:**
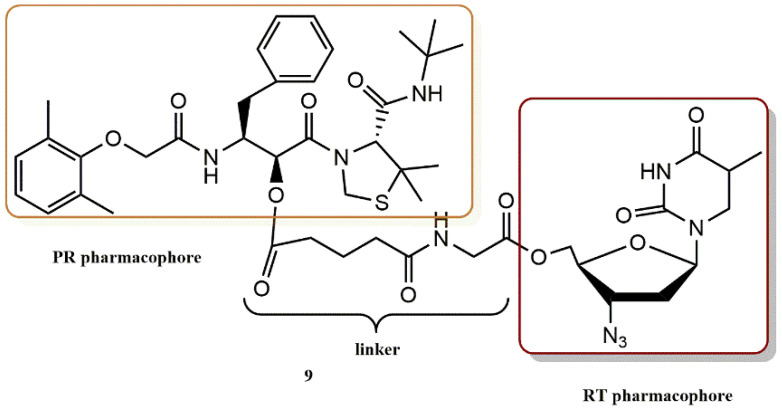
RT and PR pharmacophores of new active synthesized compound **9**.

Matsumoto et al. devised and synthesized several dual anti-HIV drugs based on the prodrug concept and the fusion of two distinct classes of anti-HIV agents [39]. Among the studied prodrugs, KNI-1039 (**9**), a “dual agent” made up of the HIV PR inhibitor KNI-727 conjugated to the RT inhibitor AZT via a glutarylglycine linker (Figure 11), demonstrated remarkable anti-HIV activity that was 920- and 62-fold stronger than that of KNI-727 and AZT, respectively. Prodrug **9** regenerated active species in the cell homogenate but was only moderately stable in the culture medium. The 5′-hydroxyl group of a nucleoside inhibitor RT, AZT, was directly esterified with the carboxyl group of HIV protease inhibitors in the produced hybrid-type anti-HIV medicines. According to these findings, the dual agent KNI-1039 (**9**) penetrates the cell membrane before regenerating active species that inhibit many targets in a synergistic manner. 

### 4.2. RT/IN Inhibition

#### 4.2.1. Pyrimidine-2,4-Diones Derivatives

**Figure 12:**
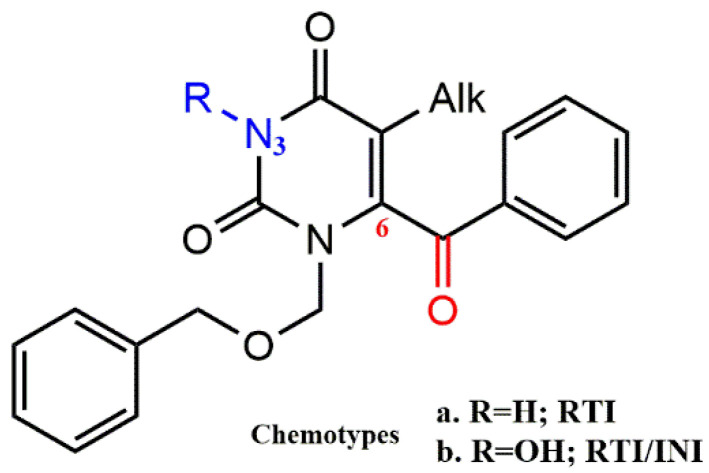
Design of the new N-3-hydroxypyrimidine-2,4-dione derivatives presenting a C-6 benzoyl group.

**Figure 13:**
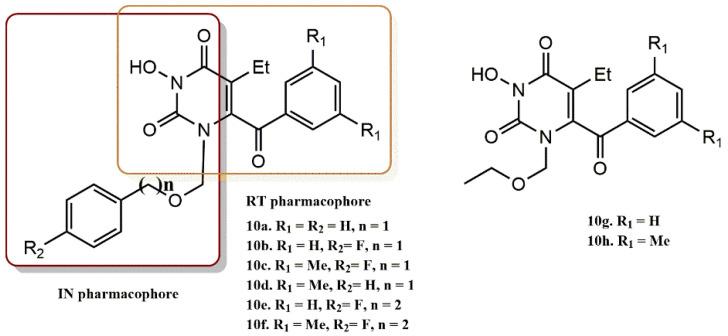
RT and IN pharmacophores of new active synthesized compounds **10a-h**.

Recently, it was discovered that N-3 hydroxylation of pyrimidine-2,4-diones generates dual HIV-1 inhibitors RT/IN. It is important to note that pyrimidine-2,4-diones with a C-6 benzoyl group have a chemical structure similar to HEPT structures and form a fundamental category of NNRT inhibitors (Figure 12, chemotype a). As shown in Figure 12 (chemotype b), the addition of an N-3 hydroxy group to these NNRTIs creates a new molecular scaffold for dual RT/IN inhibitors. In this context, Tang et al. synthesized a series of 6-benzyol-3-hydroxypyrimidine-2,4-diones with a benzoyl group at the C-6 position of the pyrimidine ring and investigated their inhibitory activity biologically (Figure 13) [40]. The dual inhibition RT/IN of the final compounds **10a-e** was evaluated. The results showed that the 3,5-dimethyl group significantly enhanced RT inhibition on the benzene ring of the C-6 substituent (**10c** vs. **10b**, **10d** vs. **10a**, **10h** vs. **10g**). However, the additional carbon in the N-1 linker (**10e** vs. **10b**, **10f** vs. **10c**) and the fluorine atom on the benzene ring of the N-1 side chain (**10b** vs. **10a**, **10c** vs. **10d**) provided a significant decrease in RT inhibition. In contrast, the same promoters benefit significantly from IN binding (**10b** vs. **10a**, **10c** vs. **10d**), and compounds **10e** and **10f** showed significantly higher IN inhibition. These results conclusively show that these compounds preferentially inhibit ST at low micromolar concentrations and also confirm the dependence of IN ST inhibition on the benzene ring in the N-1 side chain, as the two compounds (**10g** and **10h**) with an aliphatic N-1 side chain did not inhibit ST.

The binding mode of these synthesized compounds to IN and RT was investigated by molecular docking of a representative inhibitor (**10d**) at the NNRTI binding sites and the core catalytic domain of integrase (CCD). The hydrogen bond formed between N-3 OH and the K101 backbone in the NNRTI binding core resulted in a favorable conformation of the inhibitor, with the C-6 benzene ring interacting with Y188 and fitting well in the IN CCD in compound with Mg^2+^ and viral DNA. Meanwhile, the N-1 benzyl group was placed in the hydrophobic pocket at the protein–DNA interface. The two Mg^2+^ ions were chelated, corresponding to the deprotonated N-3 OH group in the pyrimidine ring. These docking results support the observed dual inhibition and indicate that this chemotype can interact with both RT and IN. This suggests that this chemotype is a novel scaffold for dual inhibition of RT/IN.

**Figure 14:**
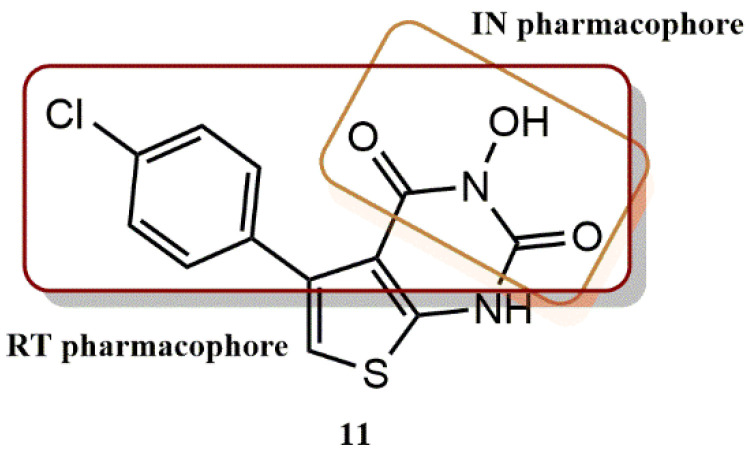
RT and IN pharmacophores of new active synthesized compound **11**.

Based on 6-phenylhydroxylisoquinoline (HID), which inhibited both HIV-1 RNase H and integrase ST at micromolar concentrations, another series of N-hydroxythienopyrimidine-2,3-dione analogs was designed and synthesized by Kankanala et al. [41]. These compounds effectively and selectively inhibited RNase H at low submicromolar concentrations and had significant efficacy against HIV-1 in cell-based assays. Although some of the analogs similarly inhibited IN-ST, this inhibition was still less than RT RNase H inhibition. The most potent compound, number **11** (Figure 14), showed low nanomolar RNase H (IC_50_ = 0.04 µM), submolecular inhibition of IN (IC_50_ = 2.1 µM), significant antiviral activity (EC_50_ = 7.4 µM), and no cytotoxicity (CC50 > 100 µM). According to the predicted binding mode of compound **11** within the RNase H binding site, the N-hydroxypyrimidine-2,4-dione (chelating triad) and the two metal cofactors (Mg^2+^) coordinated with the conserved active site residues D443, D498, D549, and E478 can potentially interact. In addition, the keto group at the 4-position of **11** appears to form H-bonds with H539, which stabilizes the position of the inhibitor near the metal-chelating residues D549, D498, D478, and D443 in the active site. These residues are most important for the inhibition of RT RNase H. 

**Figure 15:**
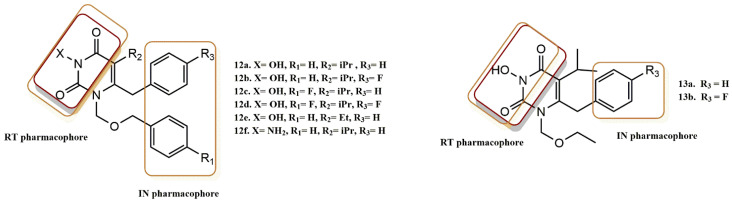
RT and IN pharmacophores of new active synthesized compounds **12a-e** and **13a-b**.

Tang et al. rationally designed another new molecular scaffold based on the NNRTI HEPT, which has N-hydroxyimide functionality that inhibits both RT and IN (Figure 15) [42]. In this design, the pre-existing benzyl group combined with a simple 3-N-hydroxylation of the pyrimidine ring of the HEPT molecule is all that is necessary to create a chelating triad and meet most of the design requirements for IN binding. This structural change did not significantly affect the compound’s potential to inhibit RT. According to the SAR analysis, the N-3 OH was critical for the inhibition of IN, and the benzyl group on the N-1 side chain was more important for IN binding than that on C-6. Specifically, a hydrophobic benzyl group and two metal ion-chelating activities are the absolute minimum requirements for integrase binding.

This design was rationally verified by docking studies. The results proved that the benzyl group at the N-1 side chain is much more crucial for binding to IN than that at the C-6 position of the pyrimidine-2,4-dione ring, as compounds **13a** and **13b** have much lower ST inhibition (10-fold) than compounds **12a-12e**. In evaluating the ability of these validated inhibitors to inhibit HIV RT, aminated compound (**12f**) and N-3-hydroxylated compound (**12c**) were both found to exhibit inhibition in low or submicromolar ranges.

N-3-hydroxylated compound (**12c**) and aminated compounds (**12f**) were all found to exhibit HIV RT inhibition in the low or submicromolar range. These compounds retain essential H-binding ability, as expected due to the presence of N-3 OH or NH_2_. Compounds **12b**, **12d**, and **13b** also show a strong fluorine effect, where a fluorine substitution at the para position of the C-6 benzyl completely prevents the binding of RT. However, the second benzyl group in the N-1 side chain appears to be more tolerant to fluorine substitution at the para position (compound **12c**). Biological studies show that the best compounds in this series, **12a** and **12c**, have low nanomolar anti-HIV activity comparable to or even higher than the reference. 

#### 4.2.2. Dihydroxyindole-2-Carboxylic Acid Derivatives

**Figure 16:**
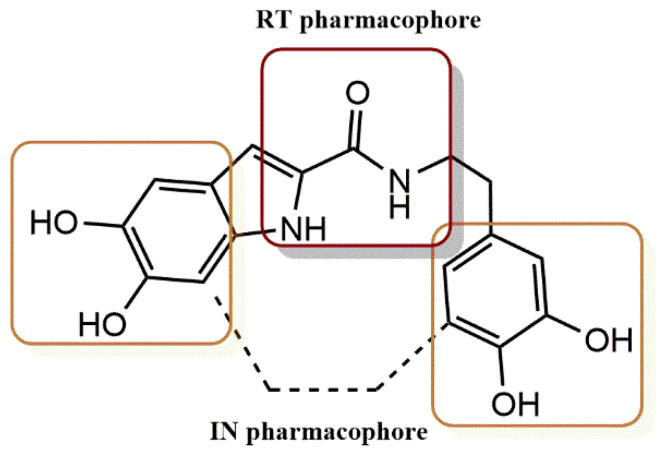
RT and IN pharmacophores of new active synthesized compound **14**.

Esposito et al. investigated the inhibitory activities of 5,6-dihydroxyindole-2-carboxylic acid (DHICA) derivatives against HIV-1 integrase and RNase H [43]. The best compound in this series was dihydroxyindole carboxamide **14** with an IC_50_ value of 1.4 μM (Figure 16). It was able to inhibit several functions of IN at low micromolar concentrations (1–18 μM). These functions included catalytic activity, IN–IN interactions, IN-LEDGF/p75 binding, and IN. Docking and site-directed mutagenesis studies have shown that compound **14** binds to a previously characterized allosteric pocket. However, these observations indicate that **14** is mechanistically and structurally distinct from previous allosteric HIV-1 IN inhibitors. In addition, compound **14** was found to be a dual HIV-1 IN and RNase H inhibitor that impairs HIV-1 replication in cell cultures because it also inhibits HIV-1 RNase H function.

#### 4.2.3. 3-Hydroxyquinazoline-2,4(1H, 3H)-Diones Derivatives

**Figure 17:**
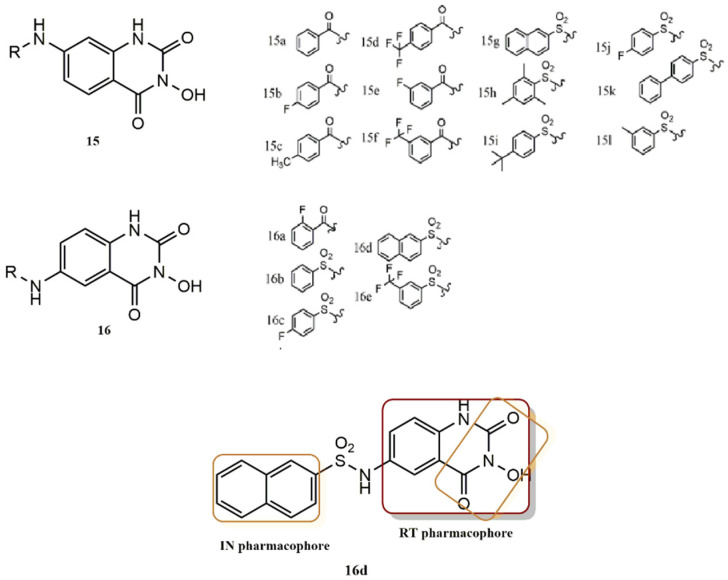
RT and IN pharmacophores of new active synthesized compounds **15a-l** and **16a-e**.

Gao et al. designed and synthesized a new series of 3-hydroxyquinazoline-2,4(1H,3H)-dione derivatives based on the hybridization of hydroXypyrimidine-2,4-dione (HPD) and hydroXylisoquinoline (HID) (Figure 17) [44]. In the development of these series, positions 6 and 7 of the quinazoline ring were modified with different benzamide or phenylsulfonamide substituents (**15**, **16**; Figure 17). An experimental study of compounds with substituents at position 7 (**15**: **15a-15l**; Figure 17) showed that the RNase H activities of derivatives with benzoyl substitutions were higher than those observed in molecules with a benzenesulfonyl substitution. Among the benzoyl-substituted compounds (**15a** to **15e**), the least substituted compound (**15a**) was the best in this series with an IC_50_ value of 2.52 ± 0.78 µM. The order of effects of the benzyl group substitutions in these compounds was: none(**15a**) > 4-methyl (**15c**) > 4-F (**15b**) 3-F (**15e**) > 4-CF_3_ (**15d**), while the efficacy of the benzenesulfonyl substitution derivatives (**17f** to **15l**) was in the following order: 2,4,6-tri-CH_3_-phenyl (**15k**), followed by 3-CH_3_-phenyl (**15l**); 3-CF_3_-phenyl (**15f**); 4-F-phenyl (**15j**); 4-tert-butylphenyl (**15i**); biphenyl (**15f**); and naphthyl (**15g**). In contrast, the compounds of series II, which were substituted with benzenesulfonyl, were more potent than the compounds substituted with benzoyl (**16a**). In addition, **16d** was less potent than raltegravir (IC_50_ = 71 ± 14 µM) but also inhibited HIV-1 IN ST (IC_50_ = 0.85 ± 0.18 µM). It is important to note that although **16d** had low cytotoxicity, its insufficient membrane permeability limited its efficacy in cell culture. The benzenesulfonyl derivatives in series II followed this order of potency: naphthyl (**16d**) > phenyl (**16b**) > 3-CF_3_ phenyl (**16e**) > 4-F phenyl (**16c**). 

A potential interaction between the chelating trio of the 3-hydroxyquinazoline-2,4(1H,3H)-dione derivative and the two Mg^2+^ ions coordinated with the conserved acidic residues Asp443, Asp498, Asp549, and Glu478 of the active site was revealed by analysis of the binding mode of the best compound (**16d**) within the active site of RNase H. Moreover, the H-bonding between His539 and the 3-hydroxyl group and the keto substituent at position 4 of **16d** is suggested as a means of stabilizing the inhibitor close to the metal-chelating residues of the active site. In contrast, the highly conserved residues (Asp128, Asp185, and Glu221) in the active site of the enzyme coordinated chelating contacts between the chemotype core of 3-hydroxyquinazoline-2,4(1H,3H)-diones and the two Mg^2+^ ions, according to the docking study of compound **16d** within the IN. Additionally, stacking interactions are formed between the DNA’s conserved terminal deoxyadenosine and the rings that contain the chelating triad of **16d**. Stacking interactions are also formed between the DNA’s conserved terminal deoxyadenosine and the rings that contain the chelating triad of **16d**. Moreover, stacking interactions between the side chain of Tyr212 and the naphthyl moiety of **16d** may occur. The IN ST inhibitory activity of **16d** seems to be supported by all of the interactions that have been characterized.

#### 4.2.4. 5-Hydroxypyrido [2,3-b]Pyrazin-6(5H)-One Derivatives

**Figure 18:**
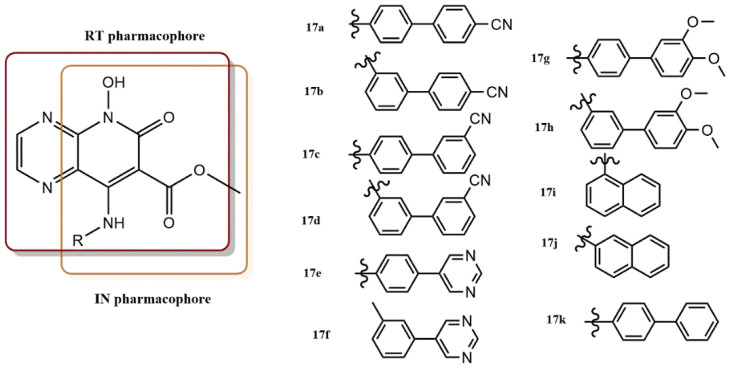
RT and IN pharmacophores of new active synthesized compounds **17a-k**.

Sun et al. reported the design, synthesis, and biological testing of a series of 5-hydroxypyrido [2,3-b]pyrazin-6(5H)-one derivatives as HIV-1 IN and RNase H dual inhibitors in the micromolar range (Figure 18) [45]. All compounds **17a-17k** were remarkably effective RNase H inhibitors with IC_50_ values ranged between 1.77 and 13.32 µM. The most active compounds in this series were compounds **17a** and **17f**, which had IC_50_ values of 1.77 and 1.85 µM, respectively, and were thus significantly more potent than the reference molecule b-thujaplicinol (IC_50_ = 2.50 µM). Notably, compound **17a** was the first reported dual HIV-1 RNase H and IN inhibitor based on a 5-hydroxypyrido [2,3-b]pyrazin-6(5H)-one structure and showed comparable inhibitory activities against RNase H and IN (IC_RNase H_ = 1.77 M, IC_IN_ = 1.18 µM, IC_RnasH_/IC_IN_ ratio = 1.50).

The experimental results and SAR analysis showed that: (i) The benzene derivatives with meta-substitution were more potent than the para-substituted benzene derivatives, as shown by **17d** and **17h**; (ii) For the para-substituted benzene derivatives, the CN position had a significant effect on the anti-RNase H potency (i.e., **17a**, **17c**), while the addition of an electron donor methoxy group had little effect on RNase H inhibitory activity (i.e., **17g** and **17k**).

The strongest analogs in this series were **17d** and **17h**, which exhibited submicromolecular inhibition activity (IC_50_ values of 0.25 and 0.22 µM, respectively) equivalent to that of RAL (IC_50_ = 0.20 µM). The 4-CN phenyl derivative (**17a**) was the most active of the para-substituted benzene analogs (**17a**, **17c**, **17e**, **17g**, and **17k**), with an IC_50_ value of 1.18 µM. The para-substituted benzene compounds were active in this order: 5-pyrimidyl (**17e**) > 4-CN phenyl derivative (**17a**) > 3,4-diOCH_3_ phenyl (**17g**), phenyl and 3-CN phenyl (**17k**).

The IN inhibitory activity of the meta-substituted benzene derivative was in the following order: 3,4-diOCH_3_ phenyl (**17h**) > 3-CN phenyl (**17d**) > 5-pyrimidyl (**17f**) > 4-CN phenyl (**17b**) and **7h** and **7d** proved to be the most potent compounds. Compounds with low IC_RNase H_/IC_IN_ ratios (~1) showed a good balance between inhibition of RNase H and IN and could be considered as effective inhibitors of both enzymes [46]. In particular, the ratio values of **17a**, **17e**, and **17j**, which were 1.50, 2.17, and 0.76, respectively, were considered as integrase/RNase H dual inhibitors.

To support the potency of compound **17a**, its binding mode within the active sites of HIV-1 IN and RNase H was predicted by molecular docking. Hydrogen bonds are formed between the hydroxyl group at the 5-position of **17a** and the carbonyl group of D498, and the nitrogen atom at the 4-position of **17a** may form another hydrogen bond with the amino group of Q500. These interactions would place **17a** near the active site. In addition, the CN group at the end of the biaryl substituent and the amino group of K540b were also found to form an additional H-bond, which is critical for the inhibition of RNase H. Moreover, the flexibility of the NH moiety at the 8-position allowed the establishment of π–π interactions between the imidazole ring of the highly conserved residue H539 and the biphenyl moiety of **17a**. The conformational flexibility of H539 is limited by this contact, which affects the catalytic potency of RNase H. The inhibition mechanism in the active site was found to be consistent with this predicted binding model.

#### 4.2.5. 3-Hydroxy-3-Phenylpropanoate Ester–AZT Derivatives

**Figure 19:**
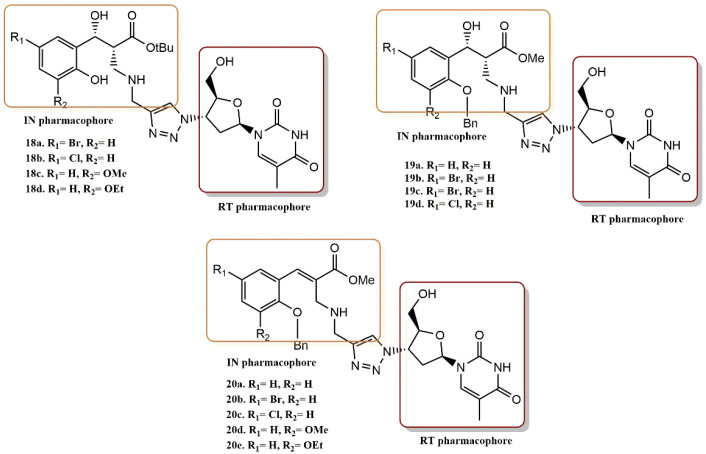
RT and IN pharmacophores of new active synthesized compounds **18a-d**, **19a-d** and **20a-e**.

Manyeruke et al. synthetized the novel 3-hydroxy-3-phenylpropanoate ester azidothymidine (AZT) series (Figure 19) [47]. Their HIV-1 RT/IN dual-inhibitory potential was investigated both experimentally (biological assays) and rationally (computer simulations). Two scaffolds with apparent HIV-1 inhibitory properties were found. Caffeic acid phenethyl ester and L-chicoric acid are specific IN inhibitors due to the presence of cinnamate ester moieties, while AZT is a proven RT inhibitor [48].

Manyeruke et al. followed up their results with SAR analysis for 3-hydroxy-3-phenylpropanoate-ester-AZT analogs. Their results are as follows:

(i) The dual inhibition RT/IN of these compounds generally followed this order: 3-hydroxy-3-phenylpropanoate ester-AZT conjugates **18a-d** > O-benzylated 3-hydroxy-3-phenylpropanoate ester-AZT conjugates **19a-d** cinnamate ester-AZT conjugates **20a-e**.
(ii) In most cases, the addition of the AZT moiety appeared to increase the percentage of IN viability, suggesting that lengthening the linker between the RT and IN active moieties may be beneficial.
(iii) The 5′-bromo-3-hydroxyl analogs **18b** and **19b** had concomitant promising potential against the enzymes RT and IN.
(iv) Most ester-AZT conjugates exhibited little or no cell toxicity.
(v) Among the sensitized derivatives, compounds (**18a**, **18b**, **18c** and **20a**) showed similar or greater inhibitory activity than their propargylated precursors with low cell toxicity (P85% cell viability at 20 µM).

The parent compound **20a** was docked to both the IN and NNRT receptor cavities and shown to form potential hydrogen bonds with amino acid residues Lys159, Lys156, Ser119, and Glu152. The ligand adopts a conformation in which the OH group is near to the Mg cofactor without distancing the metal ion, which is still complexed with the two aspartate residues. 

#### 4.2.6. Chromenone Derivatives

**Figure 20:**
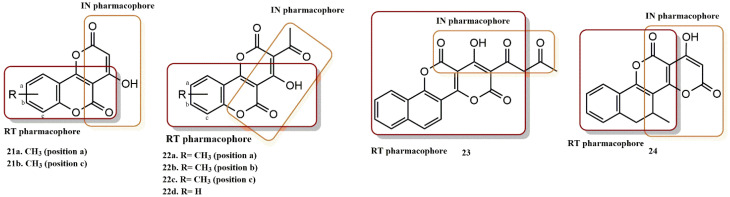
RT and IN pharmacophores of new active synthesized compounds **21a-b**, **22a-d**, **23** and **24**.

Coumarin-based compounds have already emerged as common NNRTIs targeting HIV-1 IN. These molecules block RNA-dependent DNA polymerase or DNA-dependent DNA polymerase activity and thus act as common NNRTIs. Esposito et al. used this coumarin-based scaffold to design, synthesize, and evaluate novel 4-hydroxy-2H,5H-pyrano(3,2-c)chromene-2,5-dione derivatives as new multiple HIV inhibitors (Figure 20) [49]. Molecular modeling studies were also used to predict the binding affinity of the final compounds for the active sites of HIV-1 IN and RNase H. According to the docking results, most of the synthesized derivatives fitted well into the RNase H binding site and were chelated with metal cofactor ions due to the chelating core present in their structure. Based on the interactions between the chelating cores and the two Mg^2+^ ions coordinated with the acidic residues of the binding site (Asp443, Asp498, Asp549, and Glu478), compounds **22a**, **24**, **22d**, **21a**, and **21b** were predicted to specifically bind to the RNase H active site. These residues are essential for the activity of RT RNase H. In addition, compound **22a** interacted hydrophobically with Gly444, His539, and Asn474. Compound **22c** was well-stabilized in the binding pocket via hydrophobic interactions with Gln50, central π–π interactions with His539, and coordination with the two metal cofactors.

#### 4.2.7. Diketo Acid Derivatives

**Figure 21:**
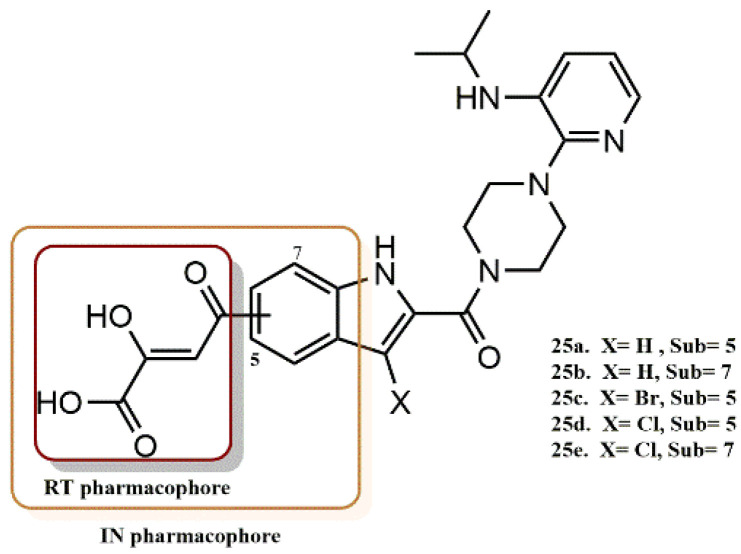
RT and IN pharmacophores of new active synthesized compounds **25a-e**.

**Figure 22:**
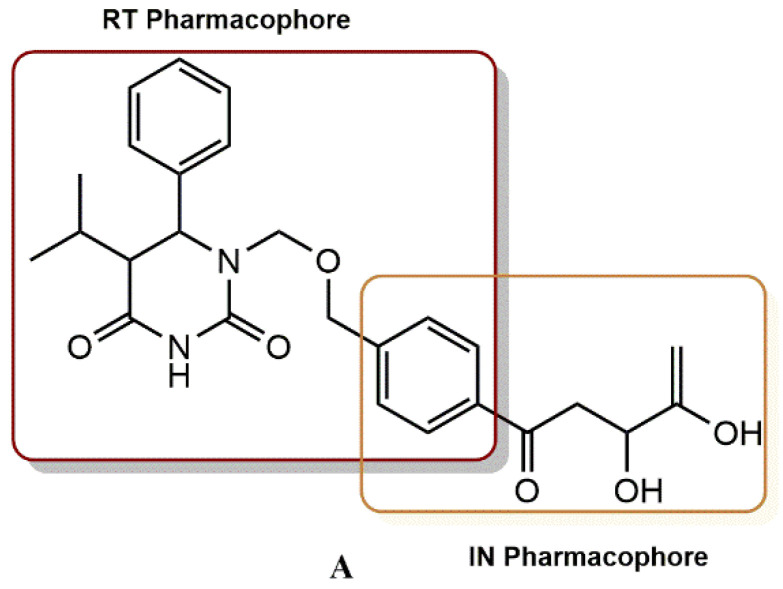
Rational design of RT/IN dual inhibitor featuring a HEPT and DKA pharmacophores for anti-RT activity and anti-IN activity, respectively.

Wang et al. rationally designed a new dual RT/IN inhibitory scaffold by introducing a DKA functionality into the C-5 site of the RT inhibitor delavirdine (Figure 21) [50]. In general, this design incorporates an IN-known pharmacophore into a proven potent RT inhibitor, as in the development of the first rationally designed RT/IN inhibitor **A** (Figure 22). The biological results showed that the C-5 substituted compounds **25a**, **25c**, and **25e** all exhibited high activity against RT and IN, while their C-7 regioisomers were all inactive. Wang et al. have also shown that balanced activities can be obtained by functionalizing the original lead compound. Inhibitor **25e** showed the greatest balance against RT (IC_50_ = 1.1 µM), IN (IC_50_ = 4.7 µM) and HIV (EC_50_ = 0.98 µM). In contrast, inhibitor **25a,** without a halogen substituent at C-3, had RT and IN activity that was separated 3-fold and similar to inhibitor **A**. The substitution position appears to have a significant effect on the binding of RT and IN. According to the modeling of RT, the DKA functionality of the compounds substituted at the C-7 position showed unfavorable electrostatic interactions with the backbone carbonyls of K103 and K104, resulting in angular molecules with almost closed conformations that might be unfavorable for binding to IN. In contrast, a delavirdine-like pseudolinear conformation is found in inhibitors with C-5 substitution, which appears to be favorable for both RT and IN binding.

#### 4.2.8. 1-[(2-Hydroxyethoxy)methyl]-6-(Phenylthio)thymine (HEPT) Derivatives

**Figure 23:**
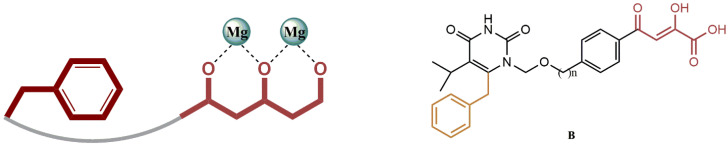
IN binding mode representation.

**Figure 24:**
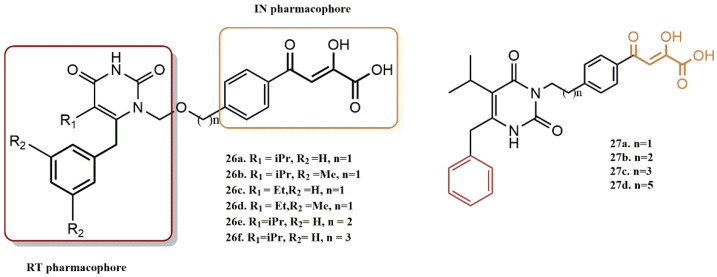
RT and IN pharmacophores of new active synthesized compounds **26a-f** and **27a-d**.

The rational design of dual HIV reverse transcriptase/integrase inhibitors based on HEPT scaffold as a proven non-nucleoside reverse transcriptase inhibitor has been shown to be feasible. To define the pharmacophores and investigate the SAR of integrase inhibition within a previously described RT/IN dual inhibitor scaffold, Wang et al. designed and synthesized new compounds with substitutions at different sites on the HEPT ring [51]. It is intriguing that the dual inhibitor (**B**) does not have the typical pharmacophore but still shows strong inhibitory activity against IN. This group hypothesizes that the C-6 benzyl group (highlighted in yellow, Figure 23) of the RT pharmacophore of HEPT could bind to the hydrophobic cavity of IN, contributing to the inhibition of IN. Figure 23 shows a detailed SAR design. To investigate the role of an additional benzyl group in integrase binding, Wang’s group first designed compounds **26a-26d** (Figure 24). By adding a benzyl group to the N-1 side chain, the right half of these molecules would resemble the conventional DKA IN inhibitor ((Z)-4-(3-benzylphenyl)-2-hydroxy-4-oxobut-2-enoic acid). The effect of the N-1 linker on the inhibition of IN was evaluated by the design of compounds **26e-26f**, while the effect of the angle between the benzyl and the chelator was investigated by the design of compounds **27a-27d**. 

Significantly, compound **26a** conferred 14-fold lower anti-HIV activity than compound (**B**), as well as reduced anti-IN (5-fold) and anti-RT (3-fold) activity. The latter is particularly intriguing because the right half of molecule **26a** is identical to the compound ((Z)-4-(3-benzylphenyl)-2-hydroxy-4-oxobut-2-enoic acid, which is a potent inhibitor of IN. It appears that the additional benzyl group in the N-1 side chain significantly reduced the IN binding affinities, strongly suggesting that only the terminal C-6 benzyl group could fit into the hydrophobic pocket, but it moved away from chelating DKA by adding this benzyl group.

These results strongly suggest that the C-6 benzyl group on the HEPT ring plays a key role in the integrase inhibition pharmacophore within the dual inhibitor scaffold. This is particularly important since the same benzyl group is also critical for reverse transcriptase inhibition. This indicates that the two pharmacophores are brought together for dual inhibition activities, which is highly sought after for dual inhibitors [52]. Moreover, extension of the N-1 linker had a negative effect on the inhibition of IN, as evidenced by the 3–4-fold decrease in anti-IN activity for compounds **26e-26f** (compared to compound **B**).

Placement of the DKA-bearing pendant at the N-3 position did not appear to prevent binding of IN, although a substantial decrease in inhibition was observed (3–15-fold for compounds **27a-27c**), with a linker containing 2–3 atoms being the most active. It was also found that the use of a linker with 6 or more carbons (compound **27d**) decreased IN inhibition. Not surprisingly, derivatization of the pyrimidine ring at N-3 (compounds **27a-27d**) resulted in a complete loss of RT activity. Thus, the proton at N-3 was found to be essential for the binding of RT, forming a potential hydrogen bonding interaction with K101 in the NNRTI binding core. Future studies will address the balance of inhibitory activity towards different enzyme targets, which can best be achieved with well-defined pharmacophores, as is the main goal of this study.

#### 4.2.9. Pyrimidine and Quinolone Derivatives

**Figure 25:**
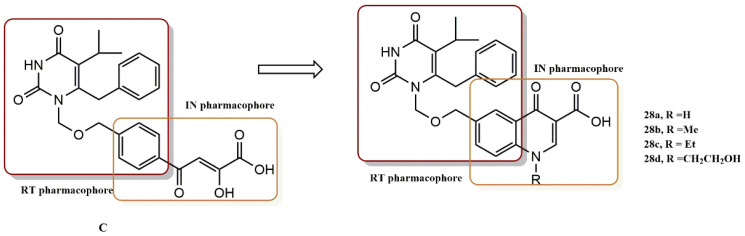
RT and IN pharmacophores of new active synthesized compounds **28a-d**.

Wang et al. designed and synthesized a series of analogs by combining pyrimidine and quinolone moieties [53]. Compound (**C**), which contains a DKA pharmacophore with dual inhibition RT/IN, served as the basis for the design. The DKA pharmacophore in compound (**C**) was replaced with an isosteric quinolone carboxylic acid moiety (**28a-28d**, Figure 25) to generate a useful scaffold for RT/IN dual inhibitors, combining a pyrimidine pharmacophore with a quinolone pharmacophore. The synthesized **28a-28d** showed activity against RT at low to submicromolar concentrations. This indicates that the addition of a second pharmacophore to the N-1 pedant of the HEPT compounds does not significantly reduce their RT binding affinity.

Furthermore, the choice of quinolonecarboxylic acid as a pharmacophore in the dual RT/IN inhibitor design was confirmed by the moderate activity observed against IN. The cell-based anti-HIV activity was comparable to the anti-RT activity, indicating that the contribution of IN to the overall activity is not significant. However, compound **28d** showed balanced anti-IN and anti-RT activity, which was better than that of the original dual inhibitor (DKA, C). Since the quinolone carboxylic acid in these conjugates only reflects the basic structural requirement for binding IN (a metal chelator with a hydrophobic aromatic ring), it is expected that the anti-IN activity can be improved with SAR analysis. It is interesting to note that all IN inhibitors included in the clinical studies have a benzyl group that is specifically related to chelating functionality.

#### 4.2.10. Madurahydroxylactone Derivatives

**Figure 26:**
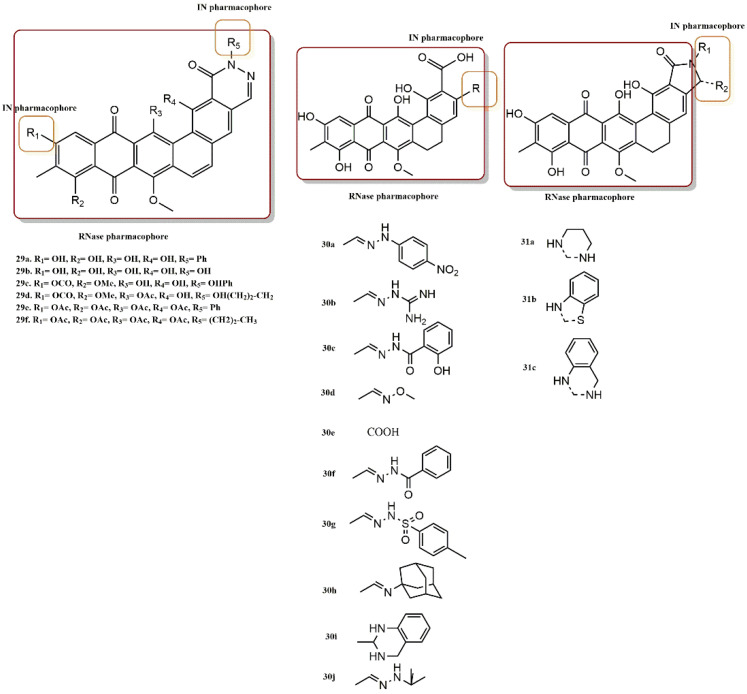
RT and IN pharmacophores of new active synthesized compounds **29a-f**, **30a-j,** and **31a-c**.

Marchand et al. evaluated a series of 29 madurahydroxylactone (MHL) derivatives for their dual HIV-1 integrase and RNase H inhibition (Figure 26) [54]. The majority of compounds showed comparable potency for both targets. However, two derivatives (**29a** and **31b**) exhibited 10- to 100-fold higher specificity for each enzyme, indicating the possibility of developing different pharmacophoric models. Twelve of the twenty-nine compounds, or nearly half, had dual activity against integrase and RNase H in the threefold range. However, with IC_50_ values generally ranging from 0.3 to 22 µM and three compounds with submicromolar IC_50_ values, all compounds inhibited HIV-1 RNase H. Compounds **30j**, **31b**, and **31c** have IC_50_ RNase H values of 0.7, 0.3, and 0.8 µM, respectively. In contrast, not all compounds inhibit HIV-1 IN. Compounds **29d**, **29e,** and **29f** showed no IN inhibition at concentrations up to 333 µM and **29a** was the best integrase inhibitor in this series (IC_50_ = 0.41 µM). Substitution of the hydroxyl group at the R_1_ position of compound **29a** with a methoxycarbonyl group completely abolished the inhibition of HIV-1 IN without affecting the inhibition of RNase H (**29a** vs. **29c**). Moreover, the presence of a phenyl ring at the R5 position of **29a** is another pharmacophoric requirement for IN selectivity. Removal of this aromatic ring leads to a 10-fold decrease in IN activity (**29a** vs. **29b**), indicating possible hydrophobic interactions between this component of the molecule and the IN residues. The chemical series **30a** to **30j** provides another structural selectivity criterion. The IN selectivity was abolished when the nitrophenyl group of the integrase-selective molecule **30a** was replaced by a phenyl ketone group of compound **30f**. The phenyl ketone group was then substituted with a t-butyl group to lead to compound **30j**, which exhibited a > 100-fold increase in RNase H selectivity. This result is also consistent with a possible hydrophobic interaction between this part of the molecule and the integrase residues. The selectivity of these compounds for RNase H was increased 40- and 20-fold, respectively, when the 1,3-piperazine ring of compound **31a** was replaced by either the phenyldiazine group of compound **31c** or the phenylthiazole group of compound **31b**. 

#### 4.2.11. Pyrrolyl Diketo Acid Derivatives

**Figure 27:**
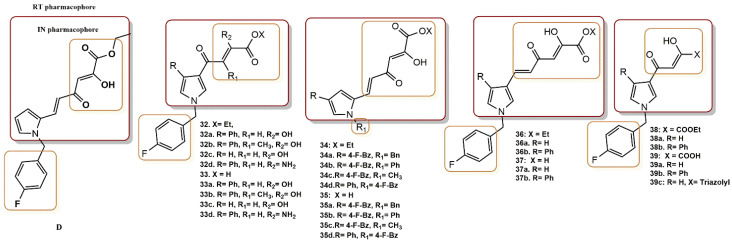
RT and IN pharmacophores of new active synthesized compounds **32a-d**, **33a-d**, **34a-d**, **35a-d**, **36a-b**, **37a-b**, **38a-b** and **39a-c**.

New pyrrolyl derivatives with good activity against IN and moderate RNase H function were designed and synthesized by Crucitti et al. [55]. To define the SAR more clearly, even in the most intriguing pyrrolyl diketoacid series, new compounds related to (D) were designed, synthesized, and biologically evaluated. Figure 27 shows all recently developed pyrrolyl DKA esters **32** and acid derivatives **33**.

The newly synthesized analogs **32a-d**, **33a-d**, **34a-d**, **35a-d**, **36a-b**, **37a-b**, **38a-b,** and **39a-c** showed good HIV-1 IN ST inhibitory activity. Acid derivatives **33a-c** were generally the most effective, as reported in previous work [56,57], with IC_50_ values in the range of 26 and 0.019 µM. In general, acid derivatives **33a-c** were the most potent, showing IC_50_ values in the range of 26–0.019 µM. Among them, seven compounds (**33a**, **33c**, **33d**, **35a**, **35d**, **39a**, **39b**) showed submicromolar activity (IC_50_ values were in the range of 66–19 µM), while two derivatives (**33(a-d)**, **35(a-d)** and **37a**) could be considered less active to almost inactive (IC_50_ values were 111 and 26 µM, respectively). The most active compound was the phenylpyrrole derivative **39b** with an IC_50_ value of 19 µM, three times less active than (D) in inhibiting the ST response of IN. Compound **39a** was confirmed as a potent IN strand transfer inhibitor with an IC_50_ value of 0.057 µM.

Among the newly synthesized pyrrolyl DKA derivatives **32(a-d)**, **34(a-d)**, **36(a-b)**,**38(a-b)**,**33(a-d)**, **35(a-d)**, **37(a-b),** and **39(a-c)**, seven derivatives (**32a**,**32c**,**38b** and **33a**,**33c**-**d**,**39b**) had good anti-HIV activities with EC_50_ values in the submicromolar range (0.56–0.9 µM) and a favorable selectivity index (SI). Three compounds, derivatives **38a-36b** and **39a**, were active in the micromolar range (1—4.3 µM), while ten compounds (**32d**, **34a-d**, **36a** and **33b**, **35e-f**, **37a**,**39c**) were less active or inactive with EC_50_ values in the range of 17.2–> 50 µM. In general, all compounds had a low cytotoxicity index (CC_50_ > 50 µM). The best dual inhibitor derivative was **34b,** which showed high activity in the micromolar range in biochemical assays but was also 20-fold less active in cell-based assays. Interestingly, among the synthesized compounds reported in this study, (**39a**) was described by Merck as the first DKA derivative selective for ST IN inhibition [58].

### 4.3. RT/RT Inhibition

#### 4.3.1. Diarylpyrimidine–Quinolone Hybrids Derivatives

**Figure 28:**
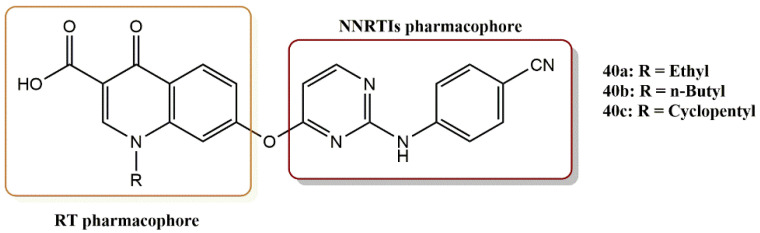
Dual RT pharmacophores of new active synthesized compounds **40a-c**.

A new series of diarylpyrimidine-quinolone hybrids was generated by Tian-Qi Mao et al. using the “molecular hybridization” strategy [59]. These hybrids with a quinolone-3-carboxylic acid residue combine the properties of RT, IN, RNase H and transcription inhibitors (Figure 28). In particular, the RNase H activities are due to the metal chelating properties of the quinolone carboxylic acid moiety. All compounds showed activity in cell-based assays and exhibited modest inhibitory effects at the micromolar level against HIV-1 RT, revealing a RT-targeted mechanism of action. The hybrids with the O-linker showed potent anti-HIV-1 activity. In particular, compounds **40a**, **40b,** and **40c** exhibited low EC_50_ values of 0.28, 0.33, and 0.98 µM and IC_50RT_ values of 7.15, 3.49, and 6.08 µM, respectively.

Compound **40b**, which was most active against RT, was docked to the non-nucleoside reverse transcriptase binding pocket (NNIBP) of HIV-1 RT. The docking results showed that these hybrids with the bulky and polar properties of a quinolone-3-carboxylic acid moiety could bind well to HIV-1 RT NNIBP. In addition, **40b** was shown to have several ligand–receptor interactions comparable to those of Entecavir (ETV), including two typical hydrogen bonds between the NH linker, Lys101, and the nitrogen atom of the pyrimidine ring. Additional π–π interactions were also observed between the quinolone ring and the hydrophobic pocket defined by the side chains of Tyr229, Phe227, Tyr188, and Tyr181.

#### 4.3.2. (3Z)-3-(2-[4-(aryl)-1,3-Thiazol-2-yl]ydrazine-1-Ylidene)-2,3-Dihydro-1H-Indol-2-One Derivatives

**Figure 29:**
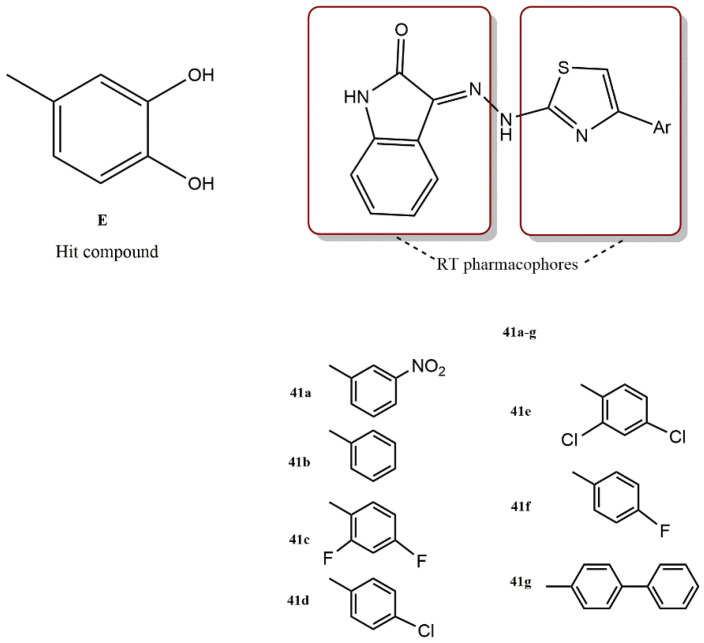
Dual RT pharmacophores of new active synthesized compounds **41a-g**.

Based on the (3Z)-3-(2-[4-(aryl)-1,3-thiazol-2-yl]ydrazine-1-ylidene)-2,3-dihydro-1H-indol-2-one scaffold, Meleddu et al. have designed and synthesized a new family of HIV-1 RT multiple inhibitors [60]. The two RT-associated functions are modulated by the properties of the aromatic group at position 4 of the thiazole ring (Figure 29). Compounds **41(a-g)** were tested for their ability to inhibit the HIV-1 RT-associated RNase H and dimerization partner (DP) activity. The results showed that all compounds except compound **41a** exhibited dual RT inhibition, confirming the scaffold (3Z)-3-(2-[4-(aryl)-1,3-thiazol-2-yl]ydrazine-1-ylidene)-2,3-dihydro-1H-indol-2-one) as a promising candidate for the development of an HIV-1 RT inhibitor with dual functions.

Interestingly, all new derivatives had a specificity index (SpI: the ratio of RNase H IC_50_ value to DP IC_50_ value) favorable for RNase H function, although compound I had an SpI favorable for DP function (SpI = 3.5). These results indicate that the nature of the substituents in position 4 of the phenylthiazole ring had a significant effect on the specificity and activity of the compound for both functions. The strongest compound (SpI of 0.11) was generated with the introduction of the 4-(4-biphenyl)thiazole moiety (**41g**). In addition, substitution with 4-(3-nitrophenyl)thiazole (**41b**) appeared to produce the most potent inhibition of DP and RNase H. However, introduction of 4-(4-biphenyl)thiazole (**41g**) or 2,4-difluorophenylthiazole (**41c**) selectively reduced the inhibition of RNase H. Thus, a balance of steric and electronic effects should be used to optimize activity toward the two RT-related functions.

Moreover, double substitution at positions 2 and 4 of 4-phenylthiazole with halogens was almost as efficient as monosubstitution. As a result, compounds **41d** and **41f** were less active than their disubstituted analogs **41e** and **41c**. In this respect, fluorine proved to be more efficient than chlorine, probably due to its higher ability to act as an acceptor for hydrogen bonds. Docking simulations were used to investigate the binding mode of compound **41g**. The allosteric binding pocket of RNase H is lipophilic and contains aromatic residues. As a result, hydrophobic interactions are critical for anti-HIV activity. The best docking position of **41g** shows a series of van der Waals interactions with Val108, Pro226, and Leu228 side chains. The biphenyl substituent was aligned with NNRTIBP and was stabilized by a variety of aromatic π–π interactions with Trp229, Phe227, and Tyr188. These stacking interactions required the presence of an aromatic ring.

#### 4.3.3. Diarylpyrimidines Derivatives

**Figure 30:**
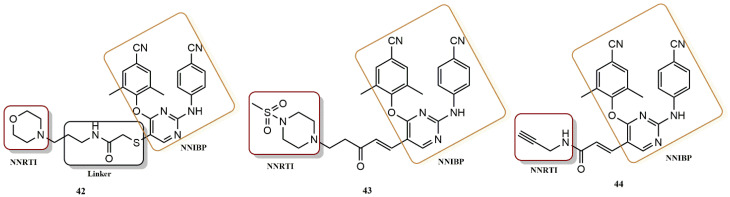
Dual RT pharmacophores of new active synthesized compounds **42**, **43** and **44**.

Feng et al. designed, synthetized, and biologically evaluated a new series of HIV-1 dual inhibitors that are diarylpyrimidine (DAPY) derivatives targeting both the adjacent NNRTI site of HIV-1 and the NNIBP (Figure 30) [61]. This series was generated by the introduction of different privileged groups (sulfonyl, acyl moietieI...) and different types and lengths of linkers were also studied. The most effective compound, according to the biological essays, was **44**, with an EC_50_ value of 2.45 nM and SI > 91, 1.5 times better than the lead compound (EC_50_ = 3.77 nM, SI > 48) and comparable to ETR (EC_50_ = 1.45 nM, SI > 158). In addition, **44** (EC_50_ = 5.2 nM) showed equivalent anti-HIV-1IIIB activity to the lead compound and ETR. Furthermore, the HIV-1 RT inhibition assay showed that compound **44** (IC_50_ = 0.0589 µM) was more effective than the reference (ETR: IC_50_ = 0.35 µM). These results support the binding of **44** to the target RT. MD simulation shows the formation of hydrogen bonds between the O atom of the amide group on linker **43** and the O atom on methylsulfonyl with highly conserved amino acid residues, Q182, and I180 at adjacent sites, respectively, to fully verify the hypothesis of the initial design. Consequently, compound **44** could serve as a new potential scaffold for the development of new inhibitors targeting both the adjacent NNRTI site and NNIBP.

#### 4.3.4. Dihydropyrimidinone-Isatin Derivatives

**Figure 31:**
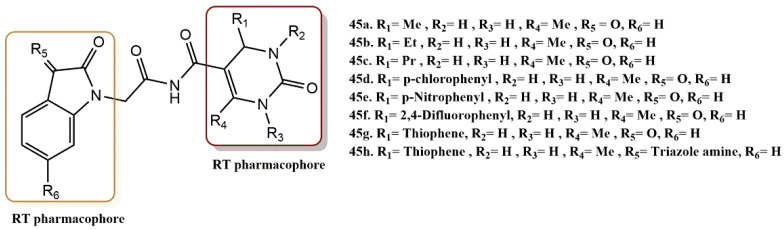
Dual RT pharmacophores of new active synthesized compounds **45a-h**.

Devale et al. combined the HEPT and isatin scaffolds to design and synthesize new anti-RT active compounds (Figure 31) [62]. After the RT inhibition experiment, it was found that six compounds (**45a-f**) had good RT inhibitory activity. In particular, compounds **45a** and **45b** were found to have RT activity better than that of the reference compound, rilpivirine. These results suggest that small aliphatic groups (such as those in **45a-c**) at the R_1_ position are more beneficial than aromatic or heteroaromatic (such as those in **45g** and **45h**) for RT inhibitory activity. Moreover, the isatin moiety may not be replaced to improve the RT activity of these hybrids. Docking analysis showed that most of the compounds exhibited a butterfly-like bond orientation, especially **45a** and **45b**. Hybrids **45a** and **45b** interacted with Lys103 (with a hydrogen bond distance of 1.61 Å and 1.86 Å, respectively) in NNIBP. Compound **45a** entered two hydrophobic π–π-stacking interactions with Tyr188 and Trp229. These interactions indicate that both the DHPM and isatin moieties were involved in the interactions with NNIBP.

#### 4.3.5. THY-ALK-TMC Derivatives

**Figure 32:**
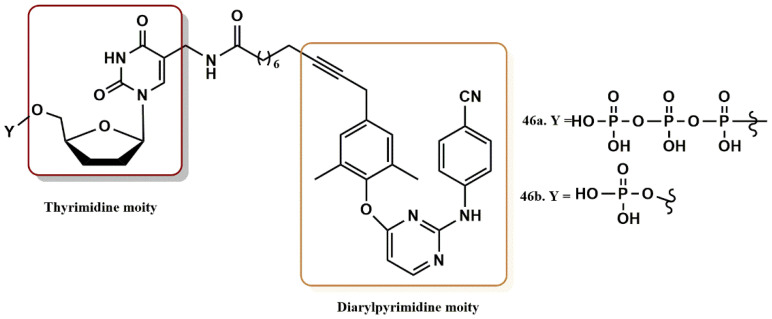
Dual RT pharmacophores of new active synthesized compounds **46a-b**.

Iyidogan et al. rationally combined thymidine (THY) and a diarylpyrimidine derivative (TMC, NNRTI) linked by a polymethylene linker (ALK) to develop a new chimeric HIV dual inhibitor (Figure 32) [63]. The goal of this study was to utilize new nucleoside components and linkers in the design (i.e., hydrophobic alkyl linkers) to better understand pharmacophores and SARs that specifically target the active site of RT polymerization and the NNIBP. Cell-based results showed that the nucleoside triphosphate derivative (**46b**) and H-phosphonate (**46a**) inhibited RT polymerization with IC_50_ values of 6.0 and 4.3 nM, respectively. These activity values were 2 and 3 times higher than those of the TMC derivative (IC_50_ = 13 nM), respectively. 

#### 4.3.6. Hydrazone Derivatives

**Figure 33:**
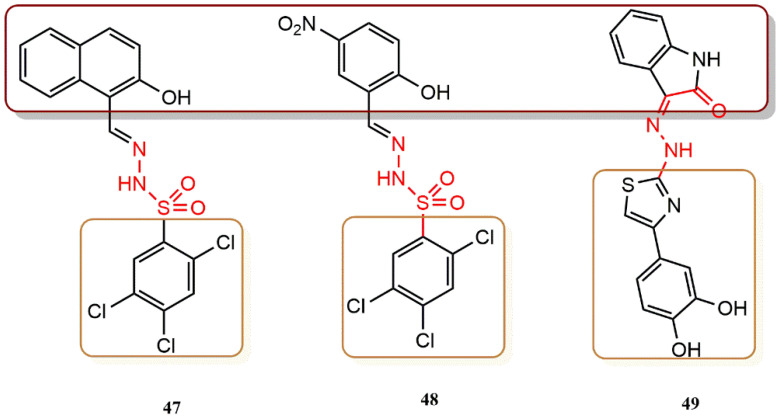
Dual RT pharmacophores of new active synthesized compounds **47**, **48** and **49**.

Distinto et al. were the first to report the use of hydrazone derivatives in silico ligand-based virtual screening (LBVS) methods in the development of new multitargeting compounds capable of inhibiting HIV-1 RT-associated functions, DNA polymerase, and RNase H activities [64]. They began with an in silico shape-based similarity screening in the NCI database and selected a hydrazone derivative previously shown to inhibit HIV-1 RT. As a result, a new scaffold was discovered: hydrazonoindolin-2-one derivative **49** (Figure 33), which offered the possibility of replacing the benzoylhydrazone moiety with a 2,4,5-trichlorobenzenesulfonehydrazone moiety (i.e., **47**, **48**, Figure 33) to yield active compounds in the low micromolar range. These new findings may pave the way for further synthesis of compounds with dual activity for both RNase H and RDDP HIV-1 RT-associated functions, leading to inhibitors that: (i) Completely prevent the activities of RT; (ii) Bind to a protein pocket that is different from the previously studied pockets; (iii) Have a new, more favorable resistance profile.

Biological analysis and docking experiments have suggested that compound **46** could bind to RT in the region suggested by Himmel et al. in previous work [65,66] and revealed the possible mode of dual activities.

## 5. Conclusions

HIV remains one of the world’s deadliest diseases more than four decades after its discovery. Several classes of anti-HIV drugs are now in use. However, viral resistance to these drugs is limiting their effectiveness in a growing percentage of people. Therefore, the development of new anti-HIV drugs with innovative treatment mechanisms is critical. The molecular hybrid strategy is an important and useful paradigm in the design and development of innovative anti-HIV therapies. These hybrid drugs can potentially slow the spread of resistance, improve patient adherence, and reduce the cost and risk of drug–drug interactions. This review focuses on the most common methods for designing and synthesizing hybrid molecules by combining two or more pharmacophore moieties with or without an appropriate linker. In this review, the therapeutic potential of these hybrid compounds and their structure–activity relationship are highlighted and reflected. Several viral proteins, including RT/RT, RT/IN, and RT/PR, have been shown to be inhibited by these compounds. 

Despite the progress made to date in synthesizing potential hybrid drugs, no anti-HIV hybrid drug is currently either in development or in preclinical trials. Synthesized hybrid drugs have shown positive results in vitro, but unfortunately, they are not active in vivo. This is mainly due to the bulky size of the synthesized hybrid compounds, which negatively affect their penetration into the cell. In addition, several studies have reported that these hybrid drugs do not achieve balanced activity against their targets. Thus, these problems need to be considered and studied in order to develop and design new hybrid anti-HIV drugs that can overcome these challenges and achieve sufficient efficacy. In our view, the choice of pharmacophore moieties, the nature of the linker, and the mechanism of action must be carefully demonstrated and justified for any hybrid drug development initiative. In addition, the advantages and disadvantages of the hybrid drug should be evaluated in comparison with current ARTs and cARTs. As a result, a variety of hybrid drugs could evolve into potential next-generation treatments for HIV infection with high inhibitory activity and low side effects.

## Figures and Tables

**Table 1:** List of cART medicines approved by the FDA for the treatment of HIV infections.

Trade Name	Generic Name (Dosage)	Target
Combivir	Lamivudine (150 mg) and Zidovudine (300 mg)	RT, RT
Trizivir	Abacavir (300 mg), Zidovudine (300 mg), and Lamivudine (150 mg)	RT, RT, RT
Kaletra	Lopinavir (80 mg) and Ritonavir ^a^ (20 mg)	CYP3A ^b^, PR
Epzicom	Abacavir (600 mg) and Lamivudine (300 mg)	RT, RT
Truvada	Emtricitabine (200 mg) and Tenofovir disoproxil fumarate (245 mg)	RT, RT
Atripla	Efavirenz (600 mg), Emtricitabine (200 mg), and Tenofovir disoproxil fumarate (245 mg)	RT, RT, RT
Complera	Emtricitabine (200 mg), Tenofovir disoproxil fumarate (300 mg), and Rilpivirine (25 mg)	RT, RT, RT
Stribild	Elvitegravir (150 mg), Cobicistat ^a^ (150 mg), Tenofovir disoproxil fumarate (245 mg), and Emtricitabine (200 mg)	IN, CYP3A ^b^, RT, RT
Triumeq	Abacavir (600 mg), Lamivudine (300 mg), and Dolutegravir (50 mg)	RT, RT, IN
Evotaz	Atazanavir (300 mg) and Cobicistat ^a^ (150 mg)	PR, CYP3A ^b^
Prezcobix	Darunavir (800 mg) and Cobicistat ^a^ (150 mg)	PR, CYP3A ^b^
Genvoya	Elvitegravir (150 mg), Cobicistat ^a^ (150 mg), Tenofovir alafenamide (100 mg), and Emtricitabine (200 mg)	IN, CYP3A ^b^, RT, RT
Odefsey	Tenofovir alafenamide (25 mg), and Emtricitabine (200 mg), Rilpivirine (25 mg)	RT, RT
Descovy	Emtricitabine (200 mg) and Tenofovir alafenamide (10 mg)	RT, RT
Juluca	Dolutegravir (50 mg) and Rilpivirine (25 mg)	IN, RT
Symfi	Efavirenz (600 mg), Lamivudine (300 mg), and Tenofovir disoproxil fumarate (300 mg)	RT, RT, RT
Symfi Lo	Efavirenz (400 mg), Tenofovir disoproxil fumarate (300 mg), and Lamivudine (300 mg)	RT, RT, RT
Cimduo	Tenofovir disoproxil fumarate (300 mg) and Lamivudine (300 mg)	RT, RT
Delstrigo	Tenofovir disoproxil fumarate (245 mg), Doravirine (100 mg), and Lamivudine (300 mg)	RT, PR, RT
Biktarvy	Bictegravir (50 mg), Tenofovir alafenamide (25 mg), and Emtricitabine (200 mg)	IN, RT, RT
Symtuza	Darunavir (800 mg), Emtricitabine (200 mg), Cobicistat ^a^ (150 mg), and Tenofovir alafenamide (10 mg)	PR, RT, CYP3A ^b^, RT
Dovato	Dolutegravir (50 mg) and Lamivudine (300 mg)	IN, RT
Cabenuva	Cabotegravir (200 mg) and Rilpivirine (300 mg)	IN, RT
Triumeq PD	Abacavir (600 mg), Lamivudine (300 mg), and Dolutegravir (50 mg)	RT, RT, IN

^a^ Pharmacokinetic enhancers used to increase the effectiveness of HIV medicine. ^b^ Antiviral drugs targeting host system.

## Data Availability

Data sharing not applicable.
